# Surgical Insight Into Paraduodenal Hernia Causing Intestinal Obstruction

**DOI:** 10.7759/cureus.83546

**Published:** 2025-05-05

**Authors:** Mari Vel, Sairam KR, Alexander Mecheri Antony, T Raghupathy, Prabanchan L G

**Affiliations:** 1 General Surgery, Sree Balaji Medical College & Hospital, Chennai, IND; 2 Surgery, Sree Balaji Medical College & Hospital, Chennai, IND

**Keywords:** abdominal ct diagnosis, acute intestinal obstruction, laparoscopic hernia repair, mesocolic hernia, paraduodenal hernia

## Abstract

Paraduodenal hernia (PDH), also known as mesocolic hernia, is a rare internal hernia resulting from a congenital anomaly caused by improper retroperitoneal fixation of the mesentery due to abnormal midgut rotation. Although uncommon, internal hernias can cause acute intestinal obstruction and pose a life-threatening risk if not promptly diagnosed and managed. This case report presents a 25-year-old male with a two-month history of progressively worsening abdominal pain, exacerbated by food intake and accompanied by vomiting. Imaging via contrast-enhanced CT revealed a left PDH, with clustering of jejunal loops and displacement of mesenteric vessels. The patient underwent successful laparoscopic surgical repair, including hernia sac reduction and defect closure. Timely identification and surgical intervention were critical in preventing complications such as intestinal perforation and peritonitis. This case underscores the clinical importance of considering PDH in patients with recurrent abdominal symptoms and highlights the benefits of early diagnosis and minimally invasive surgical management.

## Introduction

Paraduodenal hernias (PDHs) are rare congenital internal hernias that are classified as either left-sided or right-sided. They have an estimated incidence ranging from 0.2% to 0.9% in the general population and account for approximately 53% of all internal hernias [1]. Among these, left-sided PDH is more common, comprising about 75% of cases, while right-sided PDH accounts for 25% [1]. These hernias result from abnormal fixation of the midgut during embryological development, which leads to the formation of peritoneal fossae that serve as potential spaces for herniation [1,2]. The left-sided hernia occurs through the Landzert fossa, whereas the right-sided hernia involves the Waldeyer’s fossa. They can lead to intense abdominal pain, persistent gastrointestinal disturbances [2], and a range of vague or moderate symptoms such as nausea and vomiting. Due to the highly variable clinical manifestations, achieving a definitive preoperative diagnosis is often challenging. In many cases, the condition is incidentally identified during exploratory laparotomy [3,4] or may present as a rare underlying cause of small bowel obstruction, potentially resulting in bowel strangulation or perforation. If internal hernia is suspected, radiologists and surgeons must identify the orifice [5,6] and detect the marker vessels at the hernial sac's opening; these elements may assist in determining the specific form of internal hernia. We report the case of a 25-year-old male who exhibited intense abdominal pain for one day. He presented with a two-month history of increasingly intensifying abdominal discomfort, exacerbated by food consumption. A contrast-enhanced CT scan indicated the presence of an internal hernia. In the last 48 hours, the patient exhibited non-bilious vomiting containing food particles, although reported no complications with bowel motions, urination, or weight loss. The patient possessed no notable medical history and maintained a healthy lifestyle. This instance underscores the necessity for prompt detection of internal hernia in individuals experiencing recurrent abdominal pain to avert possible complications.

## Case presentation

**Figure 1:**
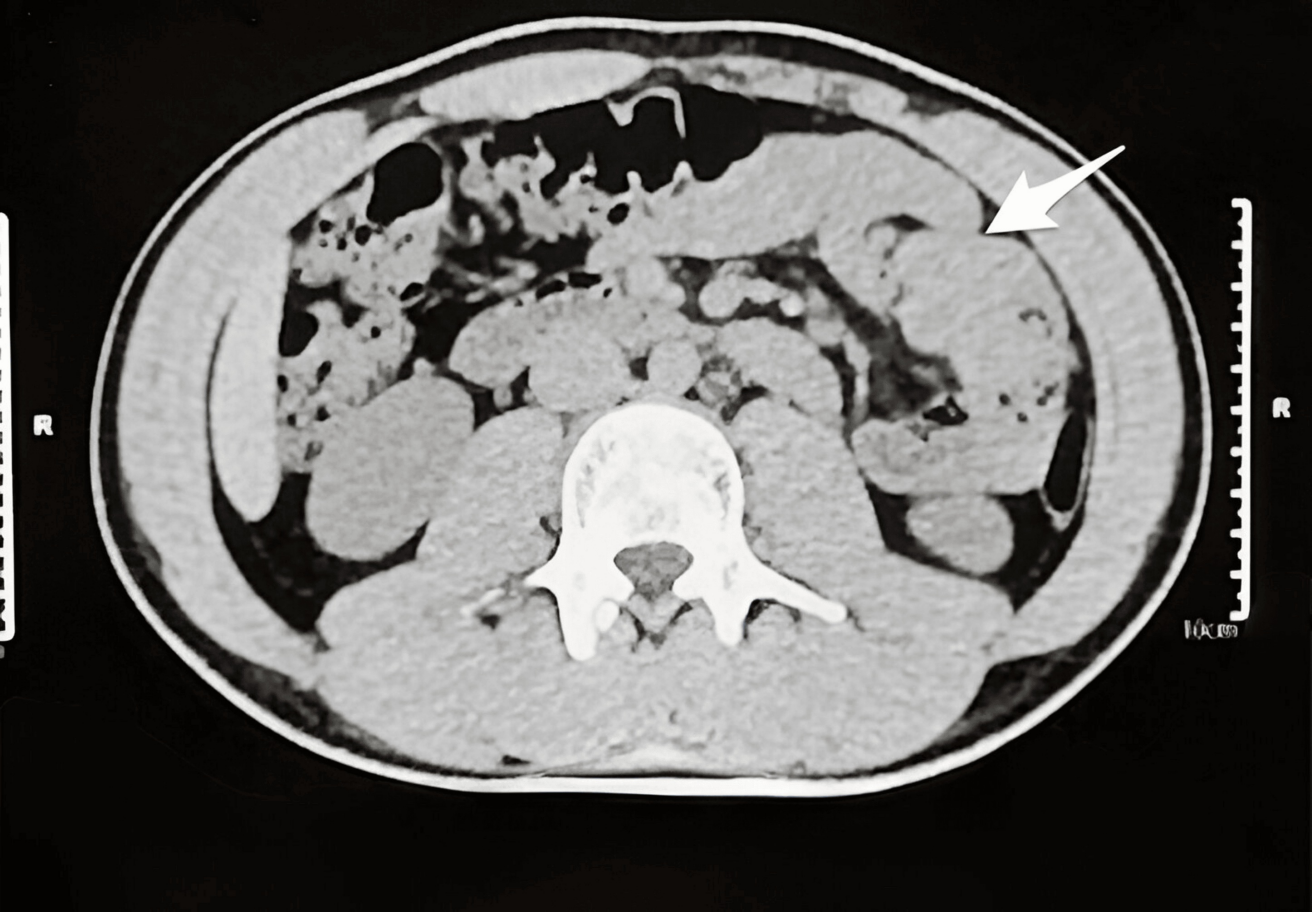
Imaging revealed a delicate, thin-walled peritoneal sac containing a cluster of small bowel loops, predominantly jejunal, situated in the left lumbar region (marked with the white arrow).

**Figure 2:**
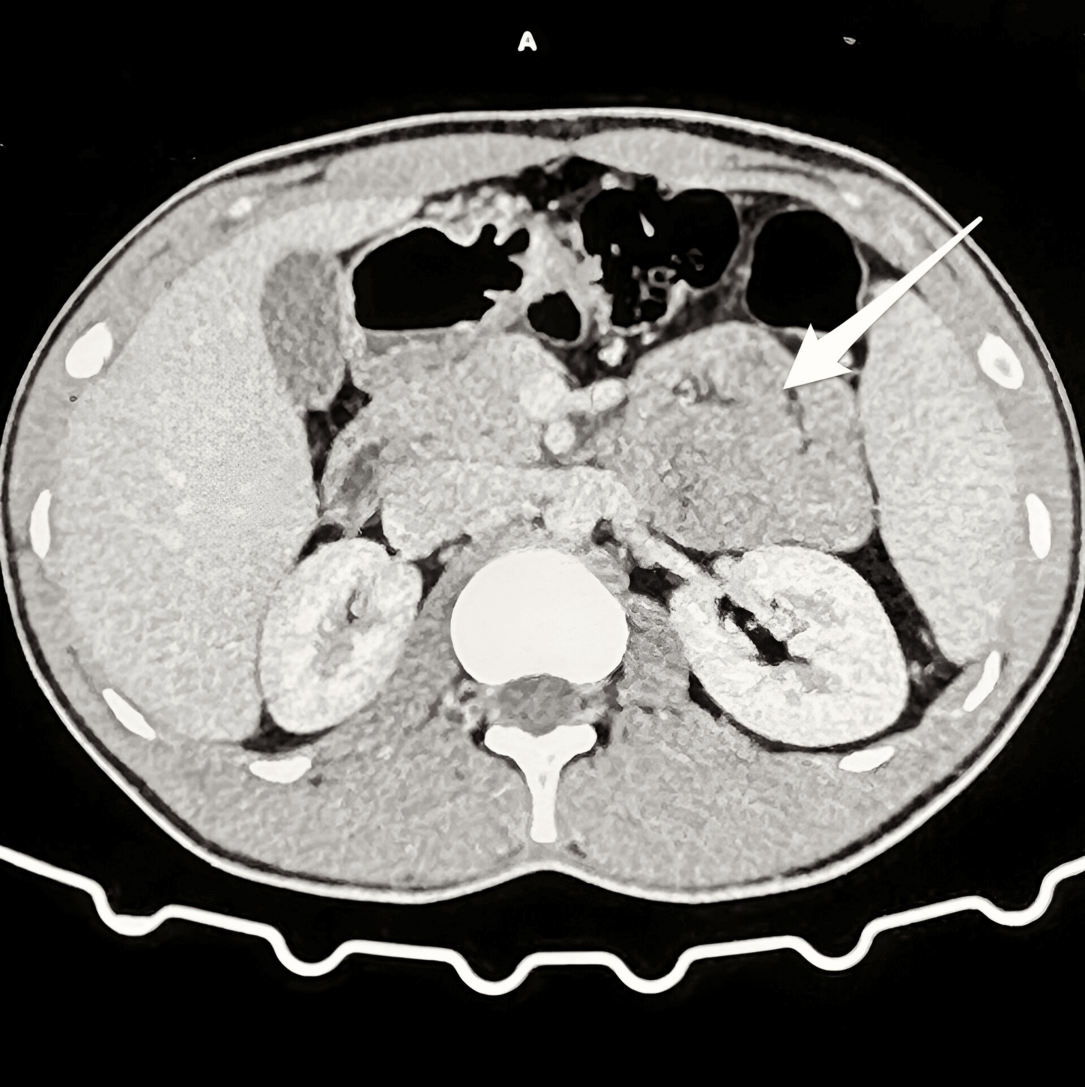
The herniated bowel loops demonstrated normal post-contrast enhancement, with mildly enlarged proximal mesenteric vessels noted (marked with the white arrow).

A 25-year-old male presented with an acute onset of abdominal pain that had persisted for one day. The pain, however, had its origins two months earlier, beginning as a diffuse and intermittent discomfort that progressively worsened over time, especially after meals. The discomfort was mildly relieved with over-the-counter medications. In the two days leading up to his hospital visit, the patient began experiencing several episodes of non-bilious, non-bloody vomiting. Despite these symptoms, he reported no signs of significant weight loss or systemic illness. His medical and surgical history were unremarkable, and no known family history of gastrointestinal diseases existed. On examination, the patient was hemodynamically stable with normal vital signs. General physical evaluation did not reveal any abnormalities. His abdomen appeared normal in contour with a centrally positioned, inverted umbilicus. There were no visible peristaltic waves, distended superficial veins, or unusual pulsations. Palpation of the abdomen did not elicit tenderness, and no masses or organomegaly was detected. There were also no palpable hernial orifices. Percussion produced areas of dullness, while auscultation revealed normal bowel sounds. A digital rectal examination confirmed normal sphincter tone, and no masses or rectal pathology was identified. Examination of the external genitalia and a systemic assessment of the cardiovascular, respiratory, and neurological systems were unremarkable. Given the persistent abdominal complaints, a contrast-enhanced computed tomography (CECT) scan was performed. The imaging revealed a left PDH with clustering of jejunal loops in the left lumbar region (Figure 1). These loops were encased within a slender peritoneal sac, which also contained the inferior mesenteric vein and the left colic artery. There was no evidence of crowding and mild twisting of the mesenteric vessels at the neck of the hernia (Figure 2). However, there were no radiologic signs of small bowel obstruction.

**Figure 3:**
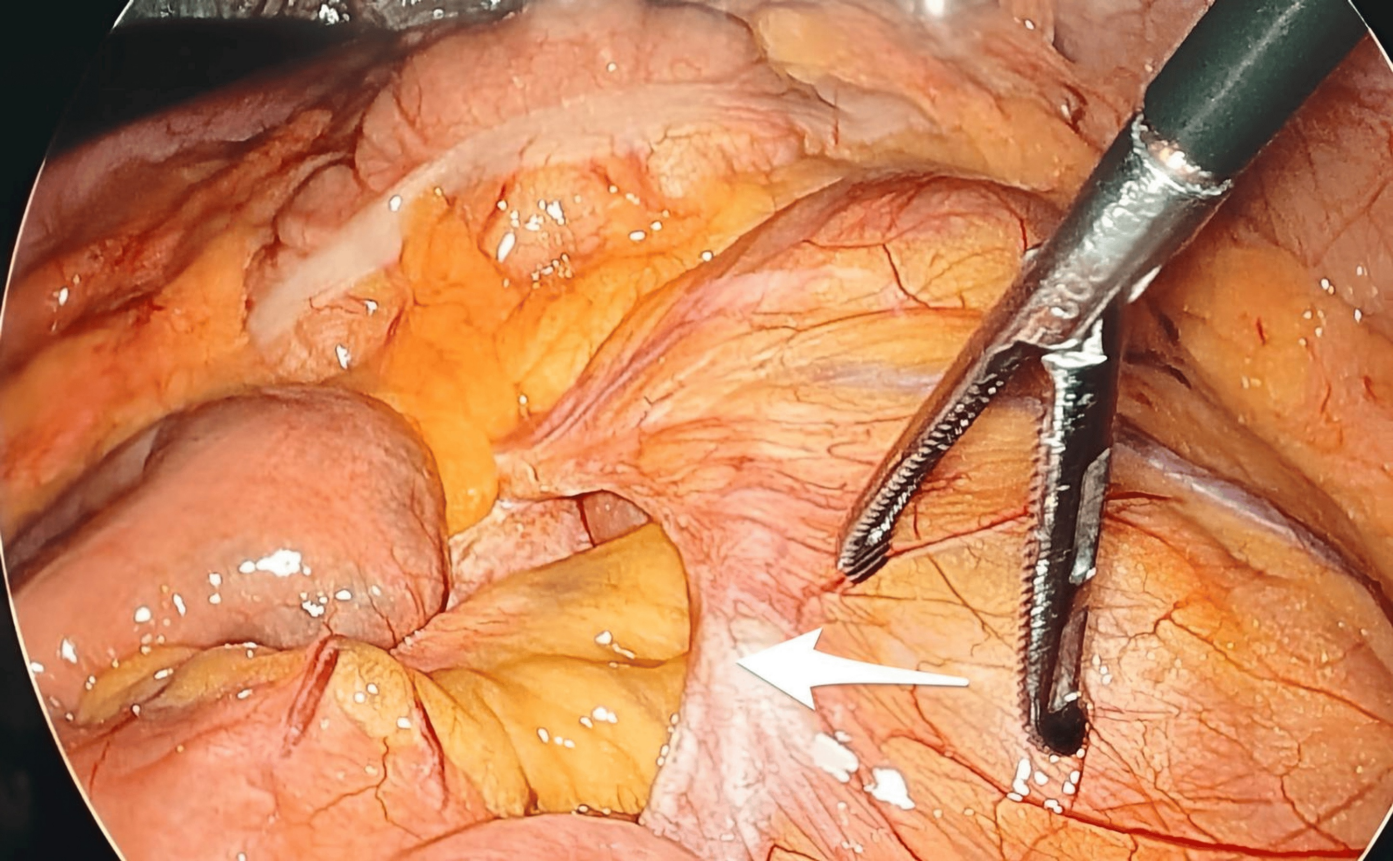
Intraoperatively, the defect was visualised, containing loops of bowel within the hernial sac (marked with the white arrow).

Following a multidisciplinary team consultation, the patient was taken for diagnostic laparoscopy. Intraoperative findings confirmed the presence of herniated jejunal loops passing through a defect adjacent to the ligament of Treitz, with the inferior mesenteric vein forming the medial boundary (Figure 3). The herniated bowel was carefully reduced, and the sac was identified. To facilitate the procedure, a Mattox maneuver was performed to mobilise the left colon. The hernial defect was then closed with 2-0 silk sutures, and an omental wrap was applied. Hemostasis was achieved, and the operation concluded without complications.

Postoperatively, the patient had an uneventful recovery and was discharged on the third day after surgery. At a one-month follow-up, he remained completely asymptomatic, with no recurrence of abdominal pain or related symptoms.

## Discussion

Internal hernias are an infrequent cause of intestinal obstruction, occurring when abdominal contents become ensnared within a compartment of the abdominal cavity. Schizas et al. [1] report that the average age of onset for PDH is 44.1 years, while Giordano et al. [2] indicate that it occurs between the ages of 40 and 60. It is thrice more prevalent in men than in women. Barium-enhanced examinations [3,7], including upper gastrointestinal series, abdominal ultrasonography, and angiography, represent additional diagnostic imaging modalities available for use. When it comes to assessing internal hernias, CECT scans and barium-based upper gastrointestinal series offer superior diagnostic accuracy [5,6] compared to other imaging techniques. Accurate preoperative identification of internal hernias through CT imaging can be enhanced by several critical factors: (a) A thorough understanding of normal peritoneal cavity anatomy [6,7] and the characteristic location associated with different types of internal hernias; (b) recognition of a cluster of dilated small bowel loops or a sac-like structure [5,8] located in an unusual region during small bowel obstruction; and (c) visualisation of an engorged, elongated, and abnormally positioned mesenteric vascular pedicle [5,6,8], often accompanied by converging blood vessels at the site of the hernia defect.

The conclusive treatment for left PDH necessitates surgical intervention, which may be executed laparoscopically or via open surgery [9,10]. The technique entails detaching the intestinal loops from the hernia sac and rectifying the defect by either suturing or extensively enlarging the hernia aperture [10], thus integrating the hernia sac into the peritoneal cavity. The laparoscopic method facilitates expedited recuperation, while the long-term outcomes are comparable.

## Conclusions

PDH typically lacks distinct symptoms and indications, presenting a diagnostic challenge that necessitates extensive knowledge and clinical acumen. The abdominal CT scan is undoubtedly the definitive diagnostic instrument. Operative repair is essential, with the laparoscopic method exhibiting considerable benefits compared to open repair, appearing to be the preferred therapeutic technique. In the presented case, the patient underwent successful laparoscopic surgery with a smooth postoperative course and was discharged on the third postoperative day. At the one-month follow-up, the patient remained asymptomatic, indicating a favorable surgical outcome.

## References

[1] Schizas D, Apostolou K, Krivan S (2019). Paraduodenal hernias: a systematic review of the literature. Hernia.

[2] Giordano G, La Mirata E, Politi V (2022). Left paraduodenal hernia in a young patient with recurrent abdominal pain: a case report and short literature review. Am J Case Rep.

[3] Martin LC, Merkle EM, Thompson WM (2006). Review of internal hernias: radiographic and clinical findings. AJR Am J Roentgenol.

[4] Newsom BD, Kukora JS (1986). Congenital and acquired internal hernias: unusual causes of small bowel obstruction. Am J Surg.

[5] Blachar A, Federle MP, Dodson SF (2001). Internal hernia: clinical and imaging findings in 17 patients with emphasis on CT criteria. Radiology.

[6] Doishita S, Takeshita T, Uchima Y (2016). Internal hernias in the era of multidetector CT: correlation of imaging and surgical findings. Radiographics.

[7] Meyers MA, Charnsangavej C, Oliphant M (2011). Meyers’ Dynamic Radiology of the Abdomen: Normal and Pathologic Anatomy. New York, NY: Springer New York.

[8] Takeyama N, Gokan T, Ohgiya Y (2005). CT of internal hernias. Radiographics.

[9] Moon CH, Chung MH, Lin KM (2006). Diagnostic laparoscopy and laparoscopic repair of a left paraduodenal hernia can shorten hospital stay. JSLS.

[10] Tong RS, Sengupta S, Tjandra JJ (2002). Left paraduodenal hernia: case report and review of the literature. ANZ J Surg.

